# Integrating hepatitis B, hepatitis C and HIV screening into tuberculosis entry screening for migrants in the Netherlands, 2013 to 2015

**DOI:** 10.2807/1560-7917.ES.2018.23.11.17-00491

**Published:** 2018-03-15

**Authors:** Janneke P Bil, Peter AG Schrooders, Maria Prins, Peter M Kouw, Judith HE Klomp, Maarten Scholing, Lutje PHM Huijbregts, Gerard JB Sonder, Toos CHFM Waegemaekers, Henry JC de Vries, Wieneke Meijer, Freke R Zuure, Alma Tostmann

**Affiliations:** 1 Department of Infectious Diseases, Public Health Service of Amsterdam, Amsterdam, the Netherlands; 2 Department of Infectious Diseases, Public Health Service of Noord- en Oost-Gelderland, Warnsveld, the Netherlands; 3 Department of Internal Medicine, Amsterdam Infection and Immunity Institute (AI&II), Academic Medical Center (University of Amsterdam), Amsterdam, The Netherlands; 4 Department of Infectious Diseases, Public Health Service of Gelderland Zuid, Nijmegen, the Netherlands; 5 Department of Medical Microbiology, Onze Lieve Vrouwe Gasthuis (OLVG), Amsterdam, the Netherlands; 6 National Coordination Centre for Communicable Disease Control, National Institute for Public Health and the Environment, Bilthoven, the Netherlands; 7 These authors share joint last authorship; 8 Department of Primary and Community Care, Radboud University Medical Center, Nijmegen, the Netherlands; Correspondence: Janneke Bil (jbil@ggd.amsterdam.nl)

**Keywords:** migrants, hepatitis B virus, hepatitis C virus, HIV infection, tuberculosis, the Netherlands

## Abstract

We evaluated uptake and diagnostic outcomes of voluntary hepatitis B (HBV) and C virus (HCV) screening offered during routine tuberculosis entry screening to migrants in Gelderland and Amsterdam, the Netherlands, between 2013 and 2015. In Amsterdam, HIV screening was also offered. Overall, 54% (461/859) accepted screening. Prevalence of chronic HBV infection (HBsAg-positive) and HCV exposure (anti-HCV-positive) in Gelderland was 4.48% (9/201; 95% confidence interval (CI): 2.37–8.29) and 0.99% (2/203; 95% CI: 0.27–3.52), respectively, all infections were newly diagnosed. Prevalence of chronic HBV infection, HCV exposure and chronic HCV infection (HCV RNA-positive) in Amsterdam was 0.39% (1/256; 95% CI: 0.07–2.18), 1.17% (3/256; 95% CI: 0.40–3.39) and 0.39% (1/256; 95% CI: 0.07–2.18), respectively, with all chronic HBV/HCV infections previously diagnosed. No HIV infections were found. In univariate analyses, newly diagnosed chronic HBV infection was more likely in participants migrating for reasons other than work or study (4.35% vs 0.83%; odds ratio (OR) = 5.45; 95% CI: 1.12–26.60) and was less likely in participants in Amsterdam than Gelderland (0.00% vs 4.48%; OR = 0.04; 95% CI: 0.00–0.69). Regional differences in HBV prevalence might be explained by differences in the populations entering compulsory tuberculosis screening. Prescreening selection of migrants based on risk factors merits further exploration.

## Introduction

In the Netherlands, it is estimated that 39,000 individuals have a chronic hepatitis B virus (HBV) infection (HBsAg-positive) [1], 19,000 have a chronic hepatitis C virus (HCV) infection (HCV RNA-positive) [2] and 23,000 have a human immunodeficiency virus (HIV) infection [3]. A large proportion of chronic HBV infections (ca 50%) and past/chronic HCV infections (ca 40%; anti-HCV positive) are estimated to be found among migrants coming from countries endemic for HBV or HCV, respectively, and ca 40% of HIV patients in clinical care are migrants [1,3,4].

Currently, effective treatment options are available for HBV, HCV and HIV infections. However, the often asymptomatic onset of these infections and disease development of HBV and HCV infections may delay diagnosis and therefore treatment. Screening for HBV, HCV and HIV can identify undiagnosed infections, improving the prognosis and limiting transmission to others by linking infected persons to treatment and care at an early stage [5-8]. To find undiagnosed cases, several HBV and HCV screening programmes, mostly community-based, have targeted specific groups of migrants in the Netherlands in recent years [9]. The prevalence found in those programmes ranged from 0% to 9.5% for chronic HBV infection (HBsAg-positive) and from 0% to 6.5% for HCV exposure (anti-HCV positive), depending on the target group and the recruitment strategy [9,10-18]. However, these programmes were not sustainable, because they were done only once, highly labour-intensive and tailored to particular migrant groups and residential areas [9]. The integration of screening into existing healthcare services could increase long-term sustainability and continuity in reaching and screening key populations. In addition, integration would most probably make such screening programmes more cost-effective because fewer additional resources would be required. As countries with high endemicity for tuberculosis (TB) largely overlap with countries with a high HBV, HCV or HIV prevalence, screening for these viruses could be integrated into the existing TB entry screening performed by TB departments of the public health services in the Netherlands. TB entry screening is compulsory for migrants from outside the European Union (EU) who intend to stay in the Netherlands for more than three months [19]. Since January 2015, the compulsory screening has been further restricted to non-EU migrants originating from countries with TB incidence of more than 50 per 100,000 inhabitants per year.

To evaluate whether integrated TB, HBV, HCV and HIV screening is effective and acceptable among migrants, we initiated a screening project offering additional voluntary HBV, HCV and HIV screening to migrants undergoing compulsory TB screening. We studied the uptake of screening and the prevalence and determinants of newly diagnosed HBV, HCV and HIV infections. The resulting data can be used to support policy-makers in the decision on integrating screening for these infections into the existing TB entry screening for migrants.

## Methods

### Study population

This screening project was performed at five TB departments of the public health services in the Netherlands (a convenience sample: four in the province of Gelderland, one in the city of Amsterdam). In Gelderland, recruitment continued until at least 352 TB department visitors had been asked to participate (October 2013 to February 2015). The sample size was based on an expected prevalence of 4.5% HBsAg-positive samples, with a 2.5% margin of error at an alpha of 0.05 in order to detect a minimum HBsAg positivity rate of at least 2%. In Amsterdam, we used a convenience sample of 250 participants, and recruitment took place from July 2015 through August 2015. Migrants visiting these TB departments have migrated primarily for work, study or family reunification. Asylum seekers are usually screened at TB departments in refugee centres and were therefore not included in this project. HBV and HCV screening was offered to all migrants attending the five TB departments for their compulsory TB entry screening. In Amsterdam, HIV screening was also offered.

### Recruitment

Migrants 18 years or older who were able to read the project information were eligible for HBV, HCV and HIV screening. In Gelderland, migrants were excluded if they had been vaccinated against HBV, whereas in Amsterdam, HBV vaccination history was not recorded. In Amsterdam, migrants were excluded if they intended to stay less than 6 months in the Netherlands, in order to ensure that those testing positive could be linked to care in the Netherlands.

All participants provided written informed consent. The project was conducted according to the ethical guidelines of the 1975 Declaration of Helsinki. The local medical ethics committee of the region Arnhem-Nijmegen (Radboud University Medical Center) approved the screening project (2013/172).

### Screening procedure

In Gelderland, migrants received information about HBV, HCV and HIV screening before their appointment for TB screening by post. In Amsterdam, where only walk-in TB consultations are provided, migrants received information about HBV, HCV and HIV screening on arrival for TB screening. Project information was available in Dutch and English and, in Amsterdam, also in Arabic, Chinese, French, Portuguese, Russian and Spanish. After eligible migrants had completed their routine TB screening, they were asked to participate in this screening project and the informed consent form was signed. Blood was drawn from those who accepted HBV, HCV and HIV screening. In Amsterdam, participants could opt out of testing for any of the three infections individually.

From all eligible migrants, the following data were collected during their TB screening visit: age, sex, country of origin and intended length of stay in the Netherlands. In Gelderland, reason for migration was included as an open-ended question. In Amsterdam, data on the reason for migration were derived from migration forms that categorised answers as work/study or other (e.g. partner or family reunification, but not further specified). In Amsterdam, participants were also asked whether they were currently registered with a general practitioner (GP) in the Netherlands and whether they had health insurance. In both regions, all persons who declined HBV, HCV and HIV screening were asked for the reason for non-participation, using an open-ended question.

### Laboratory testing

In Gelderland, blood samples were tested for anti-HBc and anti-HCV at the laboratory of the Gelre Hospital in Apeldoorn (ADVIA Centaur, Siemens, Germany), Meander Medical Center in Amersfoort (ARCHITECT, Abbott, United States) or Slingeland Hospital in Doetinchem (Cobas 6000, Roche, Switzerland). Samples positive for anti-HBc were further tested for HBsAg, anti-HBs, anti-HBe and HBeAg.

In Amsterdam, blood samples were first tested for HBsAg, anti-HCV, and HIV antigen or antibodies (LIAISON XL MUREX, DiaSorin, Italy) at the laboratory of the public health service of Amsterdam. Samples positive for HBsAg were further tested for anti-HBc, anti-HBs, anti-HBe and HBeAg. Samples positive for anti-HCV were further tested for HCV RNA (HCV Quantitative test, version 2.0, Cobas AmpliPrep/Cobas TaqMan, Roche, Switzerland). Samples positive for HIV antigen or antibodies were confirmed with Western blot (INNO-LIA HIV I/II Score, Innogenetics, Belgium), HIV-1 p24 antigen test (Vidas HIV P24, Bio Merieux) and HIV viral-load testing (HI2CAP, Roche, Switzerland).

Persons found HBsAg-positive were considered to have a chronic HBV infection. As the incidence of acute HBV infection in the Netherlands is very low, also among migrants, we assumed all HBsAg-positive persons to be chronically infected. Persons found positive for anti-HCV were considered exposed to HCV, persons positive for HCV RNA were considered to have a chronic HCV infection, and persons with confirmed HIV antigen- or antibody-positive tests were considered HIV-positive.

### Follow-up procedure

Participants who did not have a chronic HBV infection, an HCV infection or HIV infection received a letter with their test results. Participants with an infection were verbally informed of their test results by a nurse or doctor at the public health service and referred to their GP, the first point of care in the Netherlands. At the TB clinics in Gelderland, HCV RNA testing for those who tested anti-HCV positive was not included. These participants were referred to their GP for further testing. In accordance with the Dutch Public Health Act, chronic HBV infections were reported to the department of infectious diseases of the public health service in each patient’s hometown, to enable contact tracing.

In Amsterdam, participants with a chronic HBV, chronic HCV or HIV infection were contacted 3 and 6 months after they had received their results to ask if they had received follow-up care. We collected data on whether they had started treatment and whether they had visited their GP or a specialist.

### Statistical analyses

We described the following characteristics for all eligible migrants: age, sex, reason for migration, intended length of stay, region of origin, registration at GP, health insurance coverage, and HBV, HCV and HCV prevalence in the country of origin. Countries of origin were grouped into regions of origin according to the World Health Organization classification [20]. We also created three dichotomous variables (low-endemic vs intermediate/high-endemic) related to the HBV, HCV and HIV prevalence in the country of origin, using estimates by Schweitzer et al. [21], Gower et al. [22] and the Global Burden of Disease Study [23], respectively. Based on the categorisation of the reported estimates in the literature cut-off points of 2.0%, 2.5% and 2.12% were used to dichotomise HBV, HCV and HIV prevalence, respectively.

We compared the characteristics between those who refused and those who accepted the additional HBV, HCV and HIV screening, and also between participants recruited in Gelderland and those recruited in Amsterdam, using chi-squared tests for categorical variables and Mann–Whitney U test for continuous variables. We calculated the screening uptake (defined as the number of migrants who accepted screening among all the eligible persons) and described reasons for declining screening. In all analyses, the four sites in Gelderland were treated as one, as all used the same recruitment strategy and served comparable populations.

HBsAg, anti-HCV, HCV RNA and HIV prevalence and corresponding 95% confidence intervals (CI) were calculated using Wilson intervals. Using univariate logistic regression analyses, we examined determinants of a newly diagnosed chronic HBV infection, excluding persons with a previously diagnosed HBV infection. Penalised logistic regression was used to calculate odds ratios (OR) and 95% CI in a table with a zero cell count.

In all analyses, cases with unknown or missing data were excluded. Analyses were performed using STATA Intercooled 13.1 (STATA Corporation, College Station, United States). Statistical significance was set at p < 0.05.

## Results

### Characteristics of participants

A total of 968 migrants, aged 18 years or older, attended the five TB departments for their TB entry screening (Figure).

**Figure:**
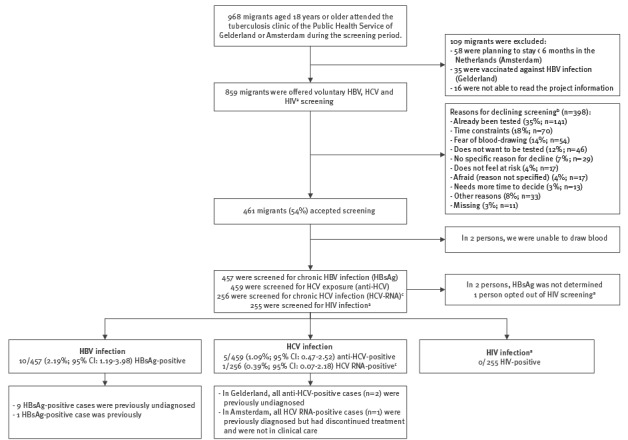
Recruitment strategy and clinical outcomes of hepatitis B, hepatitis C and HIV screening offered to migrants attending compulsory tuberculosis entry screening at the public health services, the Netherlands, 2013–2015 (n = 859)

HBV: hepatitis B virus, HCV: hepatitis C virus, HIV: human immunodeficiency virus. 

^a^ HIV screening was only offered at the Public Health Service of Amsterdam.

^b^ The percentages add up to >100% as participants could have mentioned more than one reason for declining screening.

^c^ HCV RNA was only tested in Amsterdam.

In Gelderland, 35 migrants were excluded because of prior HBV vaccination. In Amsterdam, 58 migrants were excluded because they intended to stay less than 6 months in the Netherlands. Furthermore, 16 migrants were excluded because they were unable to read the project information. Of 859 eligible migrants who were asked to participate, 461 (54%) accepted HBV or HCV (and in Amsterdam, HIV) screening. There was no significant difference between response rates in Gelderland vs Amsterdam (57% vs 51%; p = 0.113). Sex, age, region of origin, reason for migration, intended length of stay in the Netherlands and HBV and HCV prevalence in the country of origin did not significantly differ between those who refused and those who accepted screening. Participants who originated from a country with an estimated HIV prevalence of ≥ 2.12% were more likely to accept screening compared with participants from a country with an estimated HIV prevalence of < 2.12% (65% vs 52%; p = 0.022). The most commonly mentioned reasons for declining screening were: already been tested (35%; 141/398), time constraints (18%; 70/398) and fear of blood-drawing (14%; 54/398). Already been tested as a reason for declining was more likely to be reported by migrants from South-East Asia compared with other regions (50% vs 10–36%; p < 0.001) and by migrants visiting the TB clinic in Amsterdam compared with Gelderland (42% vs 25%; p < 0.001).

Two of the 461 migrants who accepted screening were ultimately not screened because blood-drawing failed. For 459 screened participants (203 in Gelderland and 256 in Amsterdam), median age was 29 years (interquartile range (IQR): 26–35 years) and 46% were male (Table 1). About half of the participants migrated for work/study (53%), and a third (34%) of all participants originated from South-East Asia. Only one person (1/256; 0.39%) in Amsterdam opted out of HIV testing, citing a low risk perception.

**Table 1:** Characteristics of migrants who accepted hepatitis B, hepatitis C and HIV^a^ screening during compulsory tuberculosis screening at public health services, the Netherlands 2013–2015 (n = 459)

	Total		Gelderland		Amsterdam		p value
	(n = 459)		(n = 203)		(n = 256)		
	**median**	**IQR**	**median**	**IQR**	**Median**	**IQR**	
Age (years)	29	26-35	28	25-34	30	27-36	< 0.001
	**n**	**%**	**n**	**%**	**N**	**%**	
Sex							
Male	211	45.97	92	45.32	119	46.48	0.804
Female	248	54.03	111	54.68	137	53.52	
Reason for migration							
Work or study	244	53.16	93	45.81	151	58.98	< 0.001
Other (e.g. family reunification)	162	35.29	110	54.19	52	20.31	
Missing	53	11.55	0	0.00	53	20.70	
Intended length of stay in the Netherlands^b^							
< 1 year	NA		19	9.36	NA		NA
1–2 years			28	13.79			
> 2 years			116	57.14			
Missing			40	19.70			
Region of origin (categorised according to WHO regions)							
South-East Asia	154	33.55	47	23.15	107	41.80	< 0.001
Europe (southern/eastern)	95	20.70	42	20.69	53	20.70	
Western Pacific	86	18.74	42	20.69	44	17.19	
Africa	61	13.29	32	15.76	29	11.33	
Eastern Mediterranean	39	8.50	22	10.84	17	6.64	
Americas (Latin America/Caribbean)	23	5.01	18	8.87	5	1.95	
Missing	1	0.22	0	0.00	1	0.39	
Estimated HBV prevalence (HBsAg-positive) in the country of origin^c^							
< 2%	204	44.44	66	32.51	138	53.91	< 0.001
≥ 2%	252	54.90	136	67.00	116	45.31	
Missing	3	0.65	1	0.49	2	0.78	
Estimated HCV prevalence (HCV-RNA positive) in the country of origin^c^							
< 2.5%	398	86.71	179	88.18	219	85.55	0.470
≥ 2.5%	60	13.07	24	11.82	36	14.06	
Missing	1	0.22	0	0.00	1	0.39	
Estimated HIV prevalence in the country of origin^c^							
< 2.12%	403	87.80	173	85.22	230	89.84	0.104
≥ 2.12%	55	11.98	30	14.78	25	9.77	
Missing	1	0.22	0	0.00	1	0.39	
Registered at a general practitioner in the Netherlands^d^							
No	NA		NA		174	67.97	NA
Yes					78	30.47	
Missing					4	1.56	
Registered for health insurance coverage in the Netherlands^d^							
No	NA		NA		72	28.13	NA
Yes, Dutch health insurance					122	47.66	
Yes, foreign health insurance					27	10.55	
Yes, student health insurance					10	3.91	
Yes, but unknown which one					21	8.20	
Missing					4	1.56	

HBV: hepatitis B virus; HCV: hepatitis C virus; HIV: human immunodeficiency virus; IQR: interquartile range; NA: not applicable (not measured); WHO: World Health Organization.

^a^ HIV screening was included only in Amsterdam.

^b^ Measured only among participants from Gelderland.

^c^ Participants were grouped and categorised according to the estimated HBV, HCV and HIV prevalence reported by Schweitzer et al. [21], Gower et al. [22] and the Global Burden of Disease Study [23], respectively.

^d^ Measured only among participants from Amsterdam.

This table excludes migrants who accepted screening but in whom blood-drawing failed (n = 2).

Participants in Gelderland were younger and more often had migrated for reasons other than work/study (e.g. family reunification) compared with Amsterdam, where most participants had migrated because of work/study. The region of origin also differed significantly between the participants from Gelderland and Amsterdam (p < 0.001). Furthermore, participants in Gelderland more often originated from a country with an HBV prevalence of ≥ 2% compared with Amsterdam participants.

### Prevalence and determinants of newly diagnosed HBV, HCV and HIV infections

In Gelderland, 29 of the 203 participants were anti-HBc-positive (14.3%; 95% CI: 10.1–19.8%) and the prevalence of chronic HBV infections was 4.48% (9/201; 95% CI: 2.37–8.29%). In two cases, HBsAg was not determined. Two of the 203 participants were anti-HCV-positive (0.99%; 95% CI: 0.27–3.52%). All HBV and HCV infections in Gelderland were newly diagnosed.

In Amsterdam, one of the 256 participants had a chronic HBV infection (0.39% (1/256; 95% CI: 0.07–2.18%). Three of 256 participants were anti-HCV-positive (1.17%; 95% CI: 0.40–3.39%) of whom one had a chronic HCV infection (0.39% (1/256; 95% CI: 0.07–2.18%). Both participants in Amsterdam with a chronic HBV and HCV infection were previously diagnosed. The participant with chronic HBV infection reported that they had been successfully treated and were being monitored in their country of origin. The participant with chronic HCV infection had started treatment in the country of origin, but discontinued it there because of side effects. This patient was referred to a Dutch hepatitis treatment centre and successfully completed HCV treatment approximately 6 months after screening. No HIV infections were found in Amsterdam.

Characteristics of all participants with a newly diagnosed chronic HBV infection (9/457; 1.97%; 95% CI: 1.04–3.70%) are shown in Table 2. In univariate analyses, participants who migrated to the Netherlands for reasons other than work/study were more likely to have a newly diagnosed chronic HBV infection than those who migrated for work/study (4.3% vs 0.8%; OR = 5.45; 95% CI: 1.12–26.60). Participants in Amsterdam were less likely to have a newly diagnosed chronic HBV infection than those in Gelderland (0% vs 4.5%; OR = 0.04; 95% CI: 0.00–0.69). No other variables were statistically significantly associated with having a newly diagnosed chronic HBV infection.

**Table 2:** Univariate analysis of potential determinants of newly diagnosed chronic hepatitis B infection among migrants who accepted hepatitis B, hepatitis C and HIV^a^ screening during compulsory tuberculosis entry screening at public health services, the Netherlands, 2013–2015 (n = 456)

	Newly diagnosed chronic HBV infection		Univariate analyses		p value
	n/N	%	OR	95% CI	
Sex					
Male	3/210	1.43	1	Ref	0.433
Female	6/246	2.44	1.72	0.43–6.98	
Age					
18–26 years	2/125	1.60	1	Ref	0.165
27–32 years	6/175	3.43	2.18	0.43–11.00	
> 32 years	1/156	0.64	0.40	0.04–4.43	
Reason for migration					
Work or study	2/242	0.83	1	Ref	0.019
Other (e.g. family reunification)	7/161	4.35	5.45	1.12–26.60	
Missing	0/53	0.00	^b ^	^b ^	
Intended length of stay in the Netherlands^c^					
< 1 year	0/19	0.00	1	Ref	0.399
1–2 years	2/28	7.14	3.68	0.17–81.03	
> 2 years	3/114	2.63	1.22	0.06–24.64	
Missing	4/40	10.00	^ b^	^ b^	
Region of origin (categorised according to WHO regions)					
South-East Asia	3/154	1.95	1	Ref	0.976
Europe (southern/eastern)	3/95	3.16	1.64	0.36–7.37	
Western Pacific	2/84	2.38	1.31	0.25–6.80	
Africa	1/60	1.67	1.09	0.16–7.56	
Eastern Mediterranean	0/39	0.00	0.55	0.28–10.83	
Americas (Latin America/ Caribbean)	0/23	0.00	0.92	0.05–18.40	
Missing	0/1	0.00	^b ^	^b ^	
Estimated HBV prevalence (HBsAg-positive) in the country of origin^d^					
< 2%	3/204	1.47	1	Ref	0.664
≥ 2%	5/249	2.01	1.37	0.32–5.82	
Missing	1/3	33.33	^ b^	^ b^	
Location of screening					
Gelderland	9/201	4.48	1	Ref	0.026
Amsterdam	0/255	0.00	0.04	0.00-0.69	

CI: confidence interval; HBV: hepatitis B virus; Ref: reference value, OR: odds ratio; WHO: World Health Organization.

^a^ HIV screening was included only in Amsterdam.

^b^ Missing categories were excluded from the analysis.

^c^ Measured only among participants from Gelderland.

^d^ Participants were grouped and categorised according to the estimated HBV prevalence reported by Schweitzer [21].

This table excludes migrants who accepted screening but in whom blood drawing failed (n = 2), participants in which HBsAg was not determined (n = 2), and the previously diagnosed HBV-infected participant (n = 1).

## Discussion

In this project, about half (54%) of the migrants attending the existing compulsory TB entry screening at public health services accepted additional HBV, HCV and HIV screening. The prevalence of chronic HBV infection (HBsAg-positive) and HCV exposure (anti-HCV-positive) in Gelderland was 4.48% and 0.99%, respectively, and all were newly diagnosed. The prevalence of chronic HBV infection in Amsterdam was 0.39%. The prevalence of HCV exposure (anti-HCV-positive) and chronic HCV infection (HCV RNA-positive) in Amsterdam was 1.17% and 0.39%, respectively. All chronic HBV and HCV infections in Amsterdam were previously diagnosed. No HIV infections were found.

Surprisingly, we found a significant difference in the prevalence of newly diagnosed chronic HBV infections between Gelderland (4.48%) and Amsterdam (0%). There are several potential explanations. The background HBV prevalence in the countries of origin of Gelderland participants was higher compared with Amsterdam participants. However, in univariate analyses, background HBV prevalence was not associated with newly diagnosed chronic HBV infection. In addition, those who migrated to the Netherlands for reasons other than work/study were more likely to have a newly diagnosed HBV infection, perhaps reflecting an increased risk among those with a lower socioeconomic status. The fact that more Gelderland participants migrated to the Netherlands for reasons other than work/study might therefore help explain the higher prevalence found among Gelderland participants. The differences in country of origin and reason for migration between participants in Gelderland and Amsterdam indicate that different areas in the Netherlands attract different groups of migrants, which is most probably due to work, study or housing opportunities in a given area or due to the migration history of family members. Another explanation for the varying prevalence of newly diagnosed chronic HBV infections might be differences in unmeasured HBV risk factors between participants from Gelderland and Amsterdam. Prior HBV vaccination was not among the exclusion criteria in Amsterdam, but it was in Gelderland.

In a comparable study from Scotland, where an integrated TB, HBV, HCV and HIV screening was only offered to international students, the prevalence of newly diagnosed HBV infections was also low (HBsAg prevalence: 2.6%, prevalence of newly diagnosed HBV infections: 1.3%, no HCV or HIV infections were found) [24]. The screening uptake found in both regions of our project was higher compared with the project in Scotland (35%) and compared with previous non-integrated HBV and HCV screening projects targeting migrants in the Netherlands (range: 7–42%) [9,10-18,24]. Uptake was lower compared with response rates for antenatal HBV and HIV screening of migrants in the Netherlands (HBV: 99.99%, HIV: 99.8%) [25], however, pregnant women could be generally more interested in screening if its primary aim is to prevent transmission to the unborn child. Furthermore, antenatal HBV and HIV testing in the Netherlands are offered according to the opt-out principle (everyone gets tested unless they explicitly refuse). The opt-out approach substantially improves HIV testing rates not only among pregnant women but also among clients of outpatient clinics focussed on sexually transmitted infections [26,27]. Similarly, an opt-out testing strategy might improve response rates to integrated HBV, HCV and HIV screening at the TB departments.

We found that the most common reason for declining screening was having already been tested. This might be indicative of a group with adequate access to care in their country of origin, in which HBV, HCV and HIV screening might therefore yield fewer newly diagnosed infections. Our results suggest that adding HIV screening is acceptable to migrants, as we saw no statistically significant difference in uptake between Amsterdam, where HIV screening was included, and Gelderland, where it was not. Only one person opted out of the HIV testing.

Unfortunately, the resources needed to add HBV, HCV and HIV screening to the compulsory TB-entry screening were not measured. Whether adding HBV, HCV and HIV screening to the compulsory TB entry screening in the Netherlands will be cost-effective needs to be further explored. A previous study from the Netherlands estimated that one-time-only, non-integrated HBV screening of all migrants from HBV-endemic countries (estimated background HBsAg prevalence: 3.35%), with a participation rate of 35%, was most probably cost-effective [28]. Although the overall HBV prevalence in our project was lower than 3.35%, overall HCV prevalence was low and no HIV infections were found, integrating HBV, HCV and HIV screening into the TB entry screening might also be cost-effective, as the programme costs of integrated screening programmes are expected to be lower compared with non-integrated screening. To further increase effectiveness, a prescreening selection of migrants based on risk factors deserves exploration, e.g. reason for migration, country of origin, or HBV, HCV and HIV risk factors such as blood transfusion history and injecting drug use [29].

If HBV, HCV and HIV screening were to be integrated into the TB entry screening, it should be taken into account that not all migrants are registered with a GP or have a Dutch health insurance at the time of screening. Although registering with a GP is easy and all Dutch citizens (including legal migrants and regardless of health status) are entitled to Dutch health insurance, extra guidance is needed to make sure that HBV, HCV and HIV-diagnosed migrants register with a GP and get a Dutch health insurance, and that they will be successfully referred and linked to specialised care [30,31]. Also, additional screening for migrants is needed to reach HBV, HCV and HIV risk groups who are not required to have TB entry screening (e.g. migrants from countries with high endemicity for HBV, HCV or HIV but with low endemicity for TB). Alternatives such as case finding through GPs should be explored for effectiveness and acceptability.

Our project has several limitations. Firstly, as our objective was to study the acceptability and effectiveness of HBV, HCV and HIV screening within the normal TB screening procedures, we decided not to measure HBV, HCV and HIV risk factors. Measuring HBV, HCV and HIV risk factors would have provided more insight into the usefulness of risk-based screening and could potentially have provided more insight into the differences between the prevalence of newly diagnosed chronic HBV infections between Gelderland and Amsterdam. Also, due to a low number of HBV infections, the analyses of demographic and migration characteristics as potential determinants of HBV were limited. Secondly, results of this project may not be generalisable to all migrants attending TB screening in the Netherlands, especially as we found regional differences in the characteristics and HBV prevalence of our populations and as migration flow changes over time. Finally, the inclusion criteria, recruitment procedures (the available translations and the timing of receipt of the project information) and screening procedure (in- or exclusion of HIV testing) differed slightly between the two regions. However, despite these small differences, uptake of screening between Gelderland and Amsterdam was similar.

## Conclusion

About half of the migrants visiting the five TB departments accepted HBV, HCV and HIV screening. The prevalence of newly diagnosed HBV infections was lower intermediate (2–4.99% [21]) in migrants screened in Gelderland, but no newly diagnosed HBV infections were found in Amsterdam. This regional difference probably reflects the differences in countries of origin and reasons for migration (which may be related to differences in social economic status) between participants in Gelderland and Amsterdam. The prevalence of newly diagnosed HCV infections was low in both regions, and no HIV infections were found. A more effective strategy might be targeted, integrated TB, HBV, HCV and HIV screening for migrants, which includes prescreening selection based on risk factors and an opt-out testing approach. Data and cost-effectiveness studies are needed for decision-making regarding the implementation of HBV, HCV and HIV screening that is integrated into entry screening at TB departments.
